# Atopic allergic conditions and prostate cancer risk and survival in the Multiethnic Cohort study

**DOI:** 10.1038/s41416-023-02364-1

**Published:** 2023-07-24

**Authors:** Anqi Wang, Peggy Wan, James R. Hebert, Loic Le Marchand, Lynne R. Wilkens, Christopher A. Haiman

**Affiliations:** 1 Center for Genetic Epidemiology, Department of Population and Public Health Sciences, Keck School of Medicine, University of Southern California, Los Angeles, CA US; 2 Cancer Prevention and Control Program, Department of Epidemiology and Biostatistics, Arnold School of Public Health, University of South Carolina, Columbia, SC US; 3 Epidemiology Program, Cancer Research Center, University of Hawaii, Honolulu, HI US

**Keywords:** Prostate cancer, Cancer epidemiology, Risk factors, Immunological disorders, Acute inflammation

## Abstract

**Background:**

Previous studies investigating relationship between atopic allergic conditions (AACs)—a highly reactive immune state—and prostate cancer (PCa) risk were inconclusive, and few have studied diverse racial/ethnic populations.

**Methods:**

We analysed 74,714 men aged ≥45 years at enrollment in Multiethnic Cohort study. Using multivariable Cox regression, we estimated hazard ratios (HRs) and 95% confidence intervals (CIs) for self-reported AAC status on PCa outcomes.

**Results:**

Through 2017, 8697 incident PCa and 1170 related deaths occurred. Twenty-one percent of men reported a history of AACs. AACs were not associated with incident PCa (HR = 0.98, 95% CI: 0.93–1.03) but were significantly inversely associated with PCa mortality (HR = 0.79, 95% CI: 0.67–0.92). This inverse association was consistently observed across all racial/ethnic groups (HR range: 0.60–0.90). Among men diagnosed with PCa, AACs were inversely associated with PCa-specific death (HR = 0.75, 95% CI: 0.63–0.89). Adjusting for potential confounding effect of PSA screening did not meaningfully change the results. No significant heterogeneity was observed in the effect of AACs on PCa incidence or mortality by Dietary Inflammatory Index.

**Conclusions:**

Hyper-allergic conditions were not associated with PCa incidence but were inversely associated with PCa mortality, suggesting a potential role in reducing tumour progression. Further aetiological research is warranted to understand underlying mechanisms.

## Introduction

Prostate cancer is the most common non-skin malignancy among men in the US and worldwide [1]. It is also one of the leading causes of cancer mortality, contributing to over 359,000 deaths per year globally [1]. Established risk factors for prostate cancer include increasing age, family history of prostate cancer, genetic predisposition, and anthropometric factors (weight and height), whereas evidence for other modifiable factors, such as environmental and lifestyle factors, is less well understood [2, 3]. Previous research suggests that the persistence of an inflammatory environment may be associated with prostate cancer development and progression [4, 5]. Adiposity, a risk factor for aggressive prostate cancer, is involved in pro-inflammatory signalling and promoting oxidative stress [6–9]. Atopic allergic conditions (AACs), such as asthma, hay fever and food allergies, are caused by an enhanced state of immune response, thereby, potentially posing an elevated risk of prostate cancer. A hyperactive immune status, alternatively, may enhance immune surveillance and have antitumour effects.

Current epidemiological evidence on the effect of AACs on prostate cancer has been mixed. The Health Professionals Follow-up Study found that men with self-reported hay fever had an increased risk of total prostate cancer, while men with asthma had a reduced risk of lethal and fatal prostate cancer [10]. By contrast, three large population-based cohort studies, the Busselton Health Survey [11], Melbourne Collaborative Cohort Study [12] and Taiwan National Health Insurance Research Database [13], reported positive associations of asthma and overall prostate cancer risk. The Busselton Health Survey also found an increased risk of prostate cancer with any atopy [11]. Two studies examined the effect of allergen-specific immunoglobulin (IgE) positivity on prostate cancer risk and reported a positive and null association, respectively [14, 15]. Apart from the mixed direction of the results, few of the aforementioned studies investigated the influence of AACs on the aggressiveness of prostate cancer [12, 16], and most studies were conducted in White populations.

Recently, the inflammatory component of diet has been suggested to modulate the chronic inflammatory process [17–19]. The Dietary Inflammatory Index (DII^®^) was developed to quantify the inflammatory potential of diet in epidemiological studies. A higher DII score represents a more pro-inflammatory diet, while a lower score represents a more anti-inflammatory composition of diet. In the last five years, emerging studies have focused on the relationship between DII and the risk of prostate cancer, and most studies have reported a positive association between a pro-inflammatory diet and total prostate cancer risk [20–28]. Given the inflammatory potential of diet, it may modify the process of prostate cancer development under chronic inflammatory conditions such as AACs.

To further understand how AACs contribute to the development of prostate cancer, in particular, aggressive and fatal prostate cancer in a diverse population, we prospectively assessed the association between AACs and prostate cancer risk in the Multiethnic Cohort (MEC) study. We also evaluated the association of AACs with prostate cancer risk and survival by race/ethnicity, as well as whether a pro-inflammatory diet, as measured by DII modifies the association.

## Methods

### Study population

The MEC is a prospective cohort study with more than 215,000 participants, primarily from five racial/ethnic groups (African-Americans, Japanese-Americans, Latinos, Native Hawaiians, and Whites), aged 45–75 years when enrolled between 1993 and 1996 in California and Hawaii [29]. Potential participants were identified through drivers’ licences and supplemented with voter’s registration files in Hawaii and the Health Care Financing Administration files in California [29]. Upon enrollment, each participant completed an extensive questionnaire regarding demographic characteristics, anthropometric measurements, personal and family history of medical conditions, medication use, lifestyle (e.g., smoking history, physical activity), and dietary intake. Specifically, a self-administrative quantitative food frequency questionnaire (QFFQ) was used to measure the frequency and quantity of intake of over 180 food items that contribute substantially to the nutrition of each ethnic group in the MEC [30, 31]. Participants were actively followed up approximately every 5 years (1998–2002, 2003–2008, 2008–2012, and 2012–2016) for updates on health conditions, uptake of screening tests for common diseases, and dietary patterns. The study was approved by the institutional review boards of the University of Hawaii and the University of Southern California.

In the current study, we included 96,888 men at baseline of the MEC in the analysis. We excluded men who did not report one of the five major racial/ethnic groups (*n* = 5941), with an implausible dietary caloric intake (*n* = 3648), with a prior prostate cancer history diagnosed before baseline (*n* = 2887), and with missing data on AAC status or other key covariates at baseline (*n* = 9698). Participants were followed from cohort entry date to the date of prostate cancer diagnosis, death, or administrative censoring on December 31, 2017, whichever was the earliest (median follow-up time = 21.5 years). Incident prostate cancer, as well as the stage (localised/regional/metastatic) and Gleason grade (low-grade [≤7]/high-grade [(≥8)) at the time of diagnosis, were ascertained by linkage to the Surveillance, Epidemiology, and End Results (SEER) statewide cancer registries in Hawaii and California. Aggressive prostate cancer was defined as either regional or metastatic disease, or, localised disease with Gleason score ≥8. Prostate cancer deaths were determined by linkage to Hawaii or California death certificate files, supplemented by the National Death Index. Survival was considered from age at diagnosis to age at death or at administrative censoring on Dec 31, 2017. In total, 74,714 men were included in the analysis and 8697 incident prostate cancer cases occurred during the follow-up, including 6559 localised, 878 regional, 438 metastatic (822 cases had missingness on stages); 5943 low grade, 2139 high grade (615 had missingness on grades); 2710 aggressive cases; and 1170 prostate cancer deaths. Only 8491 cases were included in the survival analysis because 206 cases had missing diagnosis dates, or had the same date of diagnosis as death.

### AACs and covariate assessment

AAC status was assessed at baseline based on self-report of asthma, hay fever, skin allergy, food allergy or any other allergic condition. Self-reported previous use of antihistamine for AACs (two times per week for 1 month or longer) and duration of use (≤1 year, 2–5 years, >5 years) was also assessed at baseline. The use of antihistamines and duration of use among those who reported AACs may reflect severity of the condition and were considered as an indicator of long-term heightened immune response.

We calculated the DII at baseline for each participant in the MEC as a measurement of the inflammatory potential of diet. The calculation of DII in MEC was also reported in previous studies [32, 33]. Briefly, only 28 of the 45 food components were covered in the baseline QFFQ (carbohydrate; protein; total fat; saturated, monounsaturated, and polyunsaturated fats; ω-3 and ω-6 FAs; alcohol; fibre; cholesterol; vitamins A, B-6, B-12, C, D, and E; thiamin; riboflavin; niacin; iron; magnesium; zinc; selenium; folate; β-carotene; isoflavones; and caffeine); foods not included in the MEC were dropped from the DII calculation. For each participant, a *z*-standardised score was calculated for each food component. The reference mean and standard deviation for the *z* score was derived from the dietary intake from surveys or studies conducted in 11 countries [34]. Energy-adjusted DII (E-DII) scores were calculated based on the caloric density of each food component (per 1000 kcal) to account for individual variation in overall energy intake [35]. We categorised participants into 4 groups based on the quartile distribution of E-DII in the current study population: quartile 1 (≤−2.44), quartile 2 (>−2.44 to ≤−0.88), quartile 3 (>−0.88 to ≤0.65), and quartile 4 (>0.65).

Potential confounders obtained on the baseline questionnaire included education (≤12, 13–15, ≥16 years), BMI [<18.5 (underweight), 18.5–25 (normal), 25–29.9 (overweight), ≥30 kilogram/metre^2^ (obese)], smoking status (never, former, current smokers), history of diabetes (no/yes), family history of prostate cancer (no/yes), and aspirin and/or statin use (ever use at least two times per week for 1 month or longer). PSA test utilisation history (ever/never) was first collected on the second questionnaire in 1999–2002 and 96.5% of men completed this question.

### Statistical analysis

We used Cox proportional hazards regression models of prostate cancer events with age as the time metric to estimate hazard ratios (HRs) and 95% confidence intervals (CIs) for the association between AAC status and overall prostate cancer incidence, prostate cancer severity (aggressive, low grade, high grade, localised, regional, metastatic) and prostate cancer mortality. As a potential indicator of AAC severity, we also examined the association between AAC medication use and the duration of use and prostate cancer risk. The assumption of proportional hazards was met based on the review of cumulative sums of Martingale residuals. In addition, we conducted survival analysis for men with incident prostate cancer diagnosed after baseline and examined the association between AACs and prostate cancer-specific and all-cause mortality. For all analyses, we first fitted a minimally adjusted model that included age at cohort entry and race/ethnicity, and then a fully adjusted model that included all covariates mentioned above to account for potential confounding. We additionally adjusted for stage and Gleason score as approximations for prostate cancer treatment in the survival analysis.

To address potential effect modification, we stratified models by race/ethnicity, E-DII score and PSA screening, and the likelihood ratio test was used to test for homogeneity of the effects across strata. In sensitivity analyses, PSA screening was additionally included in multivariate models to examine the potential impact of PSA screening on the relationship between AACs and prostate cancer risk.

All analyses were conducted using STATA 14.0 and R 3.6.0. A *p* < 0.05 based on a two-tailed test was considered statistically significant.

## Results

Upon enrollment, 15,630 (20.9%) men reported having been diagnosed with AACs, of whom 5481 (35.1%) reported past use of antihistamines, with 2257 (14.4%) reporting use for more than 5 years. The proportions of men who reported a history of AACs in White, African American, Native Hawaiian, Japanese American, and Latino subgroups were 24.9%, 18.5%, 21.5%, 21.5%, and 16.7%, respectively. Table 1 shows the baseline characteristic among study participants by AAC status. The mean age at enrollment was 58.57 ± 8.87 years for men with AACs and 60.03 ± 8.76 for men without AACs (*p* < 0.001). Compared to men without AACs, men with AACs were more likely to be White (31.3% vs. 25.0%), never smokers (31.5% vs. 29.7%), have a graduate college degree or higher education level (40.0% vs. 28.3%), have a family history of prostate cancer (8.2% vs. 7.2%), use aspirin and/or statins regularly (48.2% vs. 45.4%), and were less likely to have diabetes (15.5% vs. 17.8%). All the above differences were statistically significant (*p* < 0.001). The distribution of E-DII was similar between men with and without AACs (*p* = 0.26). Among 56,534 men who completed the question regarding history of PSA screening, men with AACs were more likely to report past PSA screening (50.6%) than men without AACs (44.0%, *p* < 0.001).

**Table 1:** Baseline characteristics by status of AACs in the 74,714 men in the Multiethnic Cohort study.

		Total	No AACs	With AACs	*p* value^a^
		*N* = 74,714	*N* = 59,084	*N* = 15,630	
		No. (%)	No. (%)	No. (%)	
Age at baseline (years)	≤50	15,245 (20.4%)	11,320 (19.2%)	3925 (25.1%)	<0.001
	50–60	23,248 (31.1%)	18,304 (31.0%)	4944 (31.6%)	
	60–70	26,112 (34.9%)	21,154 (35.8%)	4958 (31.7%)	
	70–80	10,109 (13.5%)	8306 (14.1%)	1803 (11.5%)	
Education level	≤8th grade	7276 (9.7%)	6307 (10.7%)	969 (6.2%)	<0.001
	9th–12th grade	22,173 (29.7%)	18,643 (31.6%)	3530 (22.6%)	
	Vocational school/some college	22,283 (29.8%)	17,398 (29.4%)	4885 (31.3%)	
	Graduated college or higher	22,982 (30.8%)	16,736 (28.3%)	6246 (40.0%)	
Ethnicity	White	19,662 (26.3%)	14,765 (25.0%)	4897 (31.3%)	<0.001
	African American	9310 (12.5%)	7588 (12.8%)	1722 (11.0%)	
	Native Hawaiian	5355 (7.2%)	4206 (7.1%)	1149 (7.4%)	
	Japanese American	23,267 (31.1%)	18,261 (30.9%)	5006 (32.0%)	
	Latino	17,120 (22.9%)	14,264 (24.1%)	2856 (18.3%)	
Smoking status	Never	22,492 (30.1%)	17,571 (29.7%)	4921 (31.5%)	<0.001
	Former	38,813 (51.9%)	30,326 (51.3%)	8487 (54.3%)	
	Current	13,409 (17.9%)	11,187 (18.9%)	2222 (14.2%)	
BMI	Underweight	482 (0.6%)	390 (0.7%)	92 (0.6%)	<0.001
	Normal	26,615 (35.6%)	20,877 (35.3%)	5738 (36.7%)	
	Overweight	34,798 (46.6%)	27,749 (47.0%)	7049 (45.1%)	
	Obese	12,819 (17.2%)	10,068 (17.0%)	2751 (17.6%)	
Diabetes history		12,923 (17.3%)	10,499 (17.8%)	2424 (15.5%)	<0.001
Family history of prostate cancer in father or brothers		5514 (7.4%)	4234 (7.2%)	1280 (8.2%)	<0.001
E-DII^b^	Quartile 1 (≤−2.44)	18,661 (25.0%)	14,678 (24.8%)	3983 (25.5%)	0.26
	Quartile 2 (−2.44, −0.88)	18,670 (25.0%)	14,841 (25.1%)	3829 (24.5%)	
	Quartile 3 (−0.88, 0.65)	18,664 (25.0%)	14,773 (25.0%)	3891 (24.9%)	
	Quartile 4 (>0.65)	18,719 (25.1%)	14,792 (25.0%)	3927 (25.1%)	
Regular use of aspirin or statin		34,338 (46.0%)	26,799 (45.4%)	7539 (48.2%)	<0.001
PSA screening history^c^		25,831 (45.7%)	19,553 (44.2%)	6278 (50.9%)	<0.001
Regular use of antihistamine for AACs		–	–	5481 (35.1%)	–

^a^*p* Value was obtained by chi-square test.

^b^Energy-adjusted Dietary Inflammatory Index.

^c^PSA screening history was limited to men who completed the second questionnaire.

**Fig. 1:**
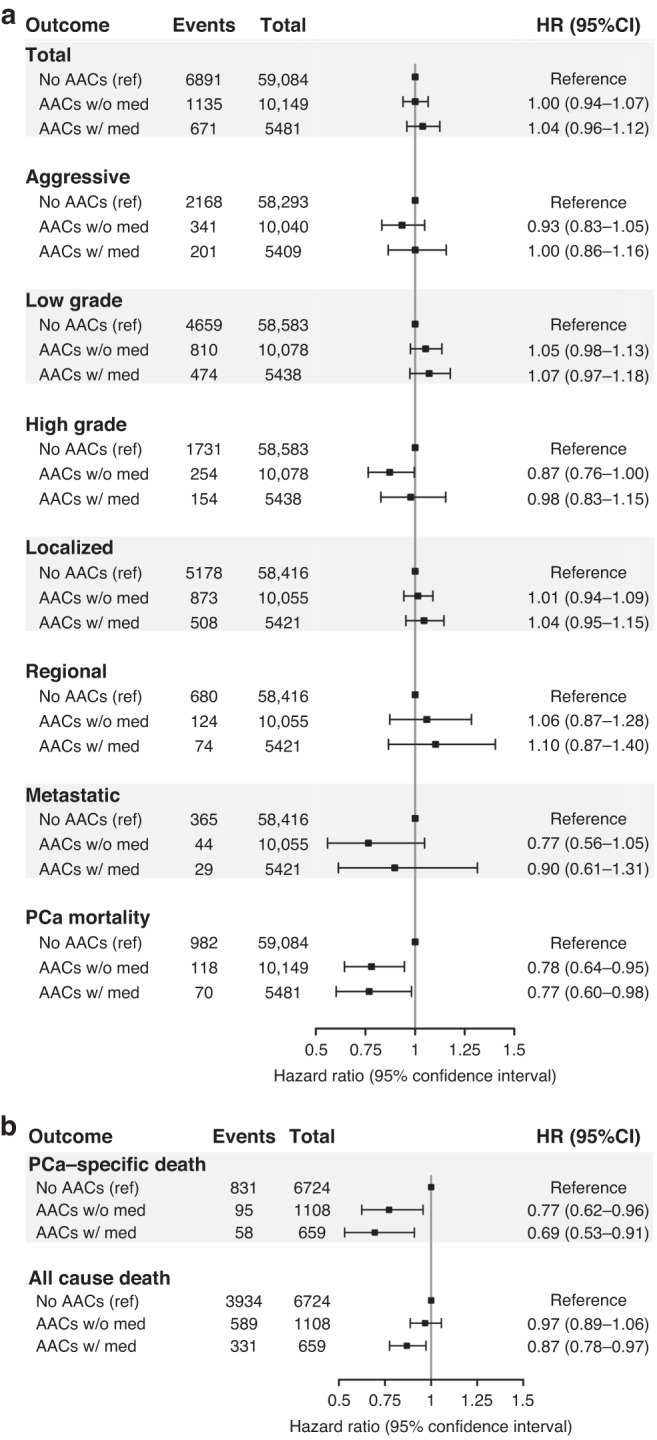
Association between antihistamine use on prostate cancer outcomes in the Multiethnic Cohort. **a** Antihistamine use and prostate cancer incidence and mortality; **b** antihistamine use and prostate cancer survival.

**Fig. 2:**
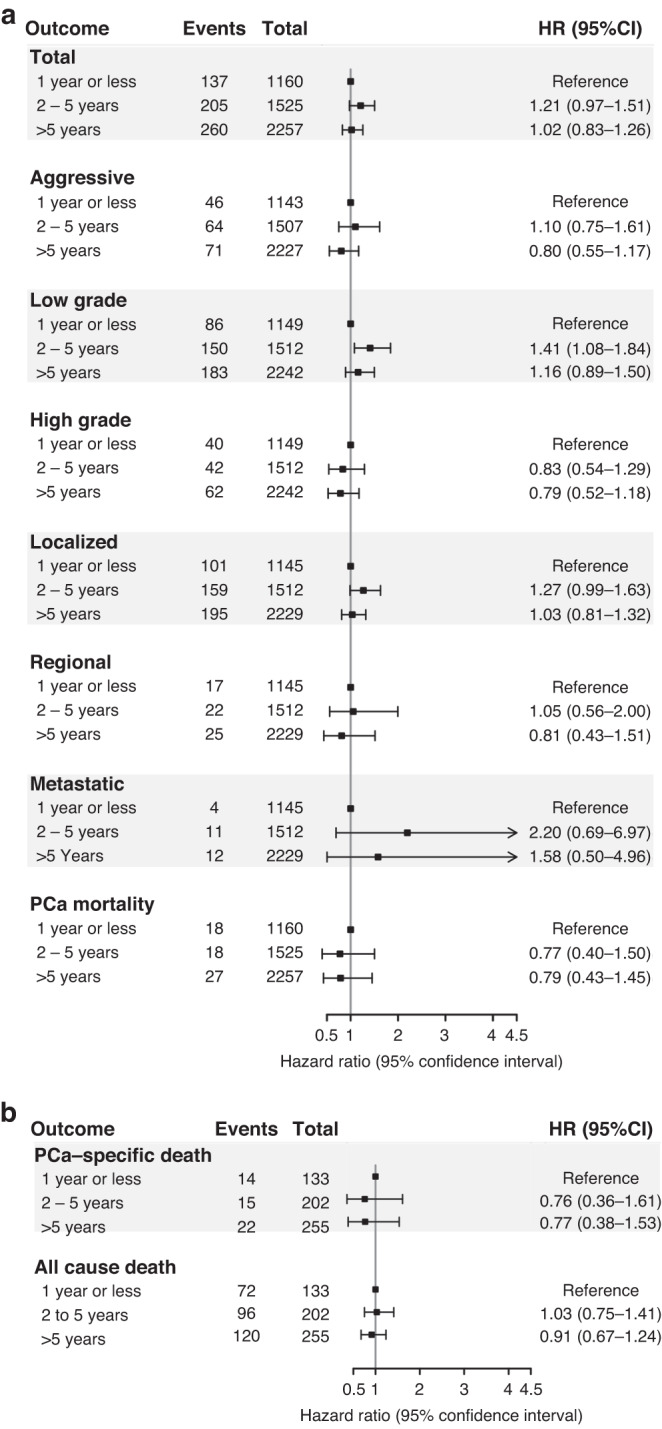
Association between the duration of antihistamine use on prostate cancer outcomes in the Multiethnic Cohort. **a** Duration of antihistamine use and prostate cancer incidence and mortality; **b** duration of antihistamine use and prostate cancer survival in the Multiethnic Cohort.

We detected no statistically significant associations between AACs and total prostate cancer incidence in the minimally adjusted model (HR = 1.00, 95% CI: 0.95–1.05) or the fully adjusted model (HR = 0.98, 95% CI: 0.93–1.03). Similarly, no association was observed between AACs and any prostate cancer subtype (Table 2). Men with and without antihistamine medication use had similar null associations with incidence of total prostate cancer or with any subtype (Fig. 1a). Among men who took antihistamines, those who reported 2–5 years of use at baseline showed a non-significant increased risk of total prostate cancer compared to those with less than 1 year of use (HR = 1.21, 95% CI: 0.97–1.51; Fig. 2a). Antihistamine use for 2–5 years was associated with significant or borderline significant increases in low grade (HR = 1.42, 95% CI: 1.09–1.85) and localised prostate cancer (HR = 1.28, 95% CI: 0.99–1.64). However, these associations were not observed in men with over 5 years of antihistamine use. E-DII was not associated with prostate cancer incidence (HR per SD = 0.98, 95% CI: 0.96–1.00), but was non-significantly associated with an increased risk of metastatic prostate cancer (HR per SD = 1.07, 95% CI: 0.97–1.18; supplemental Table 1).

**Table 2:** Hazard ratios of prostate cancer outcomes associated with AACs in the Multiethnic Cohort, 1993–2017 (*N* = 74,714).

		Events	Total	Minimally adjusted HR (95% CI)^a^	Fully adjusted HR (95% CI)^b^
Incident prostate cancer	Total	8697	74,714	1.00 (0.95,1.05)	0.98 (0.93,1.03)
	Aggressive	2710	73,742	0.97 (0.89,1.06)	0.96 (0.88,1.05)
	Low-grade	5943	74,099	1.03 (0.97,1.09)	1.01 (0.95,1.07)
	High-grade	2139	74,099	0.92 (0.83,1.02)	0.92 (0.83,1.02)
	Localised	6559	73,892	1.00 (0.94,1.06)	0.98 (0.93,1.04)
	Regional	878	73,892	1.06 (0.91,1.23)	1.04 (0.90,1.22)
	Metastatic	438	73,892	0.86 (0.68,1.07)	0.88 (0.70,1.11)
Prostate cancer mortality		1170	74,714	0.78 (0.67,0.92)	0.79 (0.67,0.92)
Prostate cancer survival^c^	Prostate cancer-specific mortality	984	8491	0.70 (0.59,0.83)	0.75 (0.63,0.89)
	All-cause death	4854	8491	0.89 (0.83,0.95)	0.93 (0.87,1.00)

^a^Adjusted for year at cohort entry and ethnicity.

^b^Adjusted for year at cohort entry, ethnicity, education, smoking, baseline BMI, diabetes, family history, and aspirin/statin intake.

^c^Additionally adjusted for prostate cancer stage and grade.

A significantly decreased risk of prostate cancer mortality was observed among men who reported a history of AACs in both minimally (HR = 0.78, 95% CI: 0.67–0.92) and fully adjusted models (HR = 0.79, 95% CI: 0.67–0.92; Table 2). The inverse association with prostate cancer mortality was observed for men who did and did not report antihistamine use (Fig. 1a).

Among men diagnosed with prostate cancer, after additional adjustment for prostate cancer stage and grade, AACs were significantly associated with a reduced risk of prostate cancer-specific mortality (HR = 0.75, 95% CI: 0.63–0.89), and a smaller reduction in risk of all-cause mortality (HR = 0.93, 95% CI: 0.87–1.00; Table 2). Moreover, compared to men without AACs, men with AACs but without antihistamine use showed a statistically significant 23% reduction (HR = 0.77, 95% CI: 0.62–0.96) in prostate cancer-specific mortality, while men with antihistamine use showed a 31% reduction in prostate cancer-specific mortality (HR = 0.69, 95% CI: 0.87–1.00) and a 13% reduction in all-cause mortality (HR = 0.87, 95% CI: 0.78–0.97; Fig. 1b). No significant associations were observed between duration of AAC medication use and prostate cancer-specific or all-cause mortality (Fig. 2b). E-DII was non-significantly associated with higher risk of prostate cancer-specific mortality and significantly associated with higher risk of all-cause mortality (HR per SD = 1.04, 95% CI: 1.01–1.07; Supplemental Table 1).

There was no significant evidence of heterogeneity in the association of AACs with prostate cancer risk or mortality by race/ethnicity (Table 3, *p* for heterogeneity >0.05 for all). An inverse association between AACs and prostate cancer-specific survival was observed in all groups (HRs < 1), but only associations among Whites and Latinos were significant (*p*s < 0.05). Similarly, we did not detect evidence of heterogeneity in the association of AACs with prostate risk or mortality by E-DII (Table 3).

**Table 3:** Hazard ratios of prostate cancer associated with AACs status among subgroups in the Multiethnic Cohort, 1993–2017.

Subgroup		Prostate cancer mortality				Prostate cancer survival (case only)				Total prostate cancer incidence							
										Prostate cancer				All cause			
		Events	Total	HR (95% CI)	*P*- het	Events	Total	HR (95% CI)	*P*-het	Events	Total	HR (95% CI)	*P*-het	Events	Total	HR (95% CI)	*P*- het
Ethnicity	White	1978	19,662	0.94 (0.85, 1.05)	0.13	313	19,662	0.79 (0.59, 1.04)	0.88	245	1907	0.71 (0.51, 0.99)	0.40	1091	1907	0.95 (0.82, 1.10)	0.37
	African American	1775	9310	1.01 (0.89, 1.14)		309	9310	0.90 (0.66, 1.21)		264	1723	0.94 (0.68, 1.31)		1172	1723	1.02 (0.87, 1.18)	
	Native Hawaiian	452	5355	1.12 (0.89, 1.40)		60	5355	0.63 (0.31, 1.30)		57	448	0.55 (0.27, 1.15)		249	448	0.72 (0.52, 1.00)	
	Japanese American	2493	23,267	0.97 (0.88, 1.07)		219	23,267	0.73 (0.50, 1.06)		193	2462	0.73 (0.49, 1.09)		1300	2462	0.90 (0.78, 1.04)	
	Latino	1999	17,120	1.13 (1.01, 1.27)		269	17,120	0.70 (0.49, 1.01)		225	1951	0.56 (0.37, 0.84)		1042	1951	0.83 (0.71, 0.98)	
E-DII^a^	Quartile 1	2405	18,661	1.06 (0.96, 1.17)	0.64	339	18,661	0.83 (0.62, 1.10)	0.50	289	2350	0.78 (0.58, 1.06)	0.07	1460	2350	0.93 (0.82, 1.06)	0.81
	Quartile 2	2269	18,670	1.03 (0.93, 1.14)		281	18,670	0.75 (0.54, 1.04)		238	2221	0.63 (0.43, 0.92)		1290	2221	0.89 (0.77, 1.03)	
	Quartile 3	2122	18,664	0.99 (0.89, 1.10)		310	18,664	0.65 (0.47, 0.90)		258	2066	0.52 (0.36, 0.77)		1133	2066	0.88 (0.76, 1.03)	
	Quartile 4	1901	18,719	0.95 (0.85, 1.07)		240	18,719	0.93 (0.67, 1.30)		199	1854	1.02 (0.72, 1.45)		971	1854	0.99 (0.84, 1.16)	
PSA screening^b^	Never	2523	30,703	0.90 (0.82, 1.00)	0.55	323	30,703	0.65 (0.47, 0.90)	0.45	248	2444	0.64 (0.43, 0.94)	0.49	1150	2444	0.91 (0.77, 1.08)	0.79
	Ever	2880	25,831	0.95 (0.87, 1.04)		361	25,831	0.81 (0.62, 1.06)		295	2808	0.80 (0.60, 1.07)		1399	2808	0.91 (0.80, 1.03)	

^a^Energy-adjusted Dietary Inflammatory Index.

^b^The date of second questionnaire completion was used as the entry time for time-to-event analysis.

Compared to men without AACs, men with AACs were more likely to report past PSA screening (OR = 1.27, 95% CI: 1.22–1.33; Supplemental Table 2), with stronger association in men reporting past AAC medication use (OR = 1.41, 95% CI: 1.32–1.50) than in men not taking medications (OR = 1.20, 95% CI: 1.14–1.26). Additionally adjusting for PSA screening history did not change any of the associations between AACs and prostate cancer risk or mortality (Supplemental Table 3). No statistically significant difference in these associations were observed when stratifying by PSA screening history (Table 3), although inverse associations were greater in men reporting no previous screening.

## Discussion

In this prospective analysis of 74,714 men from five racial/ethnic groups, AACs were not found to be associated with risk of prostate cancer. We did detect an inverse association between AACs with prostate mortality in all racial/ethnic groups that were independent of the positive association of AACs with PSA screening.

Atopic diseases generally refer to immunoglobulin E (IgE)-mediated hypersensitivity reactions to common allergens in the environment. It is estimated that approximately 8% of the US adults aged 45 years and older suffer from asthma and 11% suffer from hay fever, representing a slight increase over the past two decades [36]. The role of an allergy-related immune response in the pathogenesis and progression of prostate tumours remains unclear. Evidence suggests that in the inflammatory microenvironment of the prostate, immune cells release reactive oxygen and nitrogen species as well as pro-inflammatory cytokines that induce genetic and epigenetic mutations in normal prostate cells that could lead to abnormal epithelial cell proliferation [4, 37–40]. A hyper-reactive immune system also may have an anti-tumour effect through enhanced immunosurveillance. The cytokine profile of allergic patients is biased toward Th2 rather than Th1 cells [41]. Th2-mediated immunity activates a number of effector immune cells that are capable of recognising and attacking cancer cells at an early stage, which could result in lower prostate cancer risk and/or better survival among men with disease. It also is important to note that AACs represent Type 1 hypersensitivity involving an immediate response mediated by IgE. So, even though atopic conditions may be chronic, the biologic mechanisms involved are characterised by acute response. Indeed, it has been observed that individuals with low levels of IgE appear to be at elevated risk of cancer through mechanisms related to tumour cell phagocytosis [42]. This is consistent with proposals to use IgE as an anti-cancer therapy [43]. Mast cells, which are widely distributed in vascular tissue, are commonly associated with Type 1 hypersensitivity. These cells bind IgE with high affinity, producing TNF-α and granulocyte macrophage colony-stimulating factor (GM-CSF) in the tumour microenvironment. This observation is consistent with the idea of using tumour-targeted, IgE-sensitised mast cells as a platform for developing new cancer immunotherapies [44]. Clearly, the biologic mechanisms linking AACs and, by logical extensions, IgE is complex.

Reflecting this complexity, our finding of no association of AACs with total prostate cancer risk is in agreement with results from other studies. A meta-analysis of 20 studies (5 case–control, 15 cohort studies) through 2015 reported no significant associations for atopy (RR = 1.25, 95% CI: 0.74–2.10), hay fever (RR = 1.04, 95% CI: 0.99–1.09) or any allergy (RR = 0.96, 95% CI: 0.86–1.06) [45]. Another meta-analysis that combined results from 14 studies (7 case-control, 7 cohort studies) through 2015, also concluded an overall null association between asthma and prostate cancer risk (OR = 0.99, 95% CI: 0.84–1.18) [46]. Similarly, a Mendelian randomisation study based on the meta-analysis of GWAS summary statistics also noted no evidence of genetic predisposition to AACs and prostate cancer risk (OR = 1.00, 95% CI: 0.94–1.05) [47]. The absence of an association may reflect a dynamic balance of immunosurveillance against tumour cells and tumour-promoting immune responses.

Prostate cancer mortality and prostate cancer-specific survival were inversely associated with AACs. The relationship between AACs and prostate cancer mortality or severity has been rarely examined in epidemiological studies. Similar to our findings, the Health Professionals Follow-up Study reported significant protective effects of asthma on lethal (RR = 0.71, 95% CI: 0.51–1.00) and fatal (RR = 0.64, 95% CI: 0.42–0.96) prostate cancer [10]. This is consistent with IgE-related hypotheses noted earlier [43, 44]. Other studies examined asthma and/or allergies have shown no significant association with aggressive prostate cancer [12, 48] or prostate cancer mortality [49]. The higher survival rate among men with AACs may imply a stronger immune surveillance role in the tumour microenvironment after prostate cancer onset. This is supported by the observation in our study that AACs were not associated with localised or low-grade prostate cancer, whereas they were inversely associated with risk of disease progression (i.e., metastatic disease), and is consistent with the use of IgE in cancer treatment. This suggests that a hyper-allergic state may not prevent disease development at early stages, but may prevent tumour progression and spread to nearby tissues.

In comparison with other studies, our study has several strengths. First, this population-based prospective study included a diverse racial and ethnic population, which results in more generalisable findings. Second, we were able to investigate the impact of PSA screening. Men with AACs are more likely to undergo PSA screening than their non-affected counterparts, which may allow for increased detection of prostate cancer, although we did not find a positive association between AACs and disease risk. The positive association between AACs and PSA screening may also result in early detection, downstaging of disease at the time of diagnosis, and treatment leading to a better survival. However, the results remained robust after adjusting for and stratifying by PSA screening history. Third, this is also the first study to examine and report on inflammatory components of diet and the lack of effect modification on the association between AACs and prostate cancer risk.

Despite its strengths, several limitations of our study should also be noted. First, we did not have information on the specific types of AACs in our study, and consequently we were only able to study the combined effect of all types of AAC. All AACs we evaluated were Type 1 hypersensitivities, characterised by exaggerated IgE-mediated immune responses, which allowed for an assessment of the collective impact of enhanced IgE activity on prostate cancer risk. However, these conditions may not present identical immunologic effects and their associations with prostate cancer could vary. In addition, we were unable to ascertain the onset and duration of AAC, factors which could potentially influence prostate cancer incidence and mortality. Second, we did not enquire about the complete list of AAC-related medications, such as glucocorticoids, which have been found to be associated with prostate cancer incidence [12, 48]. However, we expect systemic glucocorticoids to play a relatively small role in explaining the inverse relationship between AACs and prostate cancer mortality, as few patients with AACs take glucocorticoids. Third, PSA screening history was only collected at a single time point, detailed information on timing and frequency of PSA screening test was not available. The PSA screening effect might not be fully accounted for in the PSA screening-adjusted and stratified analyses. Finally, as in any large prospective cohort, we are reliant on participant self-report for a wide range of variables, including those related to AAC symptomology and diet. These reports could be subject to a variety of information biases.

In summary, we observed an inverse association between AACs and prostate cancer mortality across White, African-American, Native Hawaiian, Japanese-American, and Latino men that was independent of the effect of PSA screening. Further aetiological research on the relationship between allergic response and prostate cancer progression, particularly by type, onset, and duration of AAC, as well as mechanistic research to further biological understanding is warranted.

## Supplementary information

Supplemental tables

## Data Availability

The data sets generated during and/or analysed during the current study are not publicly available due to privacy policies and data use agreements to protect participant confidentiality but are available from the corresponding author on reasonable request.
