# Supplementary material for: Atopic allergic conditions and prostate cancer risk and survival in the Multiethnic Cohort study
Source: Br J Cancer. 2023 Jul 24;129(6):974–81. doi: 10.1038/s41416-023-02364-1 (PMC10491765; doi:10.1038/s41416-023-02364-1)
Supplement: Supplementary file 1 — Supplemental tables [file 41416_2023_2364_MOESM1_ESM.pdf]

Atopic allergic conditions and prostate cancer risk and survival in the Multiethnic Cohort Study

Supplemental Tables

Supplemental Table 1. Hazard Ratios of Prostate Cancer Outcomes Associated with e-DII in the Multiethnic Cohort, 1993-2017 (N=74,714)

			Quartiles of e-DII				P value for Trend[b]	Per SD increase in e-DII	
			**Q1**	**Q2**	**Q3**	**Q4**		**HR (95%CI)**	**P-value**
**Incident prostate cancer [a]**	**Total**	**Total, No.**	18661	18670	18664	18719		74714	
		**Events, No.**	2405	2269	2122	1901		8697	
		**HR (95%CI)**	1 [Reference]	0.98 (0.93,1.04)	0.98 (0.92,1.04)	0.96 (0.90,1.02)	0.18	0.98 (0.96,1.00)	0.12
	**Aggressive**	**Events, No.**	774	683	653	600		2710	
		**HR (95%CI)**	1 [Reference]	0.93 (0.84,1.03)	0.94 (0.85,1.05)	0.94 (0.84,1.05)	0.26	0.97 (0.94,1.01)	0.20
	**Low grade**	**Events, No.**	1616	1570	1465	1292		5943	
		**HR (95%CI)**	1 [Reference]	0.99 (0.92,1.06)	0.97 (0.90,1.04)	0.92 (0.85,0.99)	0.03	0.97 (0.94,1.00)	0.03
	**High grade**	**Events, No.**	630	536	506	467		2139	
		**HR (95%CI)**	1 [Reference]	0.93 (0.83,1.04)	0.95 (0.84,1.07)	0.97 (0.85,1.10)	0.62	0.98 (0.94,1.02)	0.35
	**Localized**	**Events, No.**	1866	1709	1585	1399		6559	
		**HR (95%CI)**	1 [Reference]	0.97 (0.90,1.03)	0.96 (0.90,1.03)	0.93 (0.86,1.00)	0.05	0.97 (0.94,0.99)	0.01
	**Regional**	**Events, No.**	222	213	229	214		878	
		**HR (95%CI)**	1 [Reference]	0.91 (0.75,1.10)	0.96 (0.79,1.16)	0.89 (0.73,1.09)	0.36	1.00 (0.93,1.07)	0.91
	**Metastatic**	**Events, No.**	112	114	101	111		438	
		**HR (95%CI)**	1 [Reference]	1.10 (0.84,1.43)	1.07 (0.81,1.40)	1.30 (0.99,1.71)	0.09	1.07 (0.97,1.18)	0.17
**Prostate cancer mortality [a]**		**Total, No.**	18661	18670	18664	18719		74714	
		**Events, No.**	339	281	310	240		1170	

		**HR (95%CI)**	1 [Reference]	0.91 (0.78,1.07)	1.14 (0.97,1.33)	1.03 (0.87,1.23)	0.27	1.04 (0.98,1.11)	0.19
**Prostate cancer survival [a]**	**Prostate cancer-specific mortality**	**Total, No.**	2350	2221	2066	1854		8491	
		**Events, No.**	289	238	258	199		984	
		**HR (95%CI)**	1 [Reference]	0.93 (0.78,1.10)	1.12 (0.95,1.34)	1.05 (0.87,1.27)	0.29	1.05 (0.98,1.12)	0.15
	**All-cause death**	**Events, No.**	1460	1290	1133	971		4854	
		**HR (95%CI)**	1 [Reference]	1.02 (0.95,1.10)	1.02 (0.95,1.11)	1.13 (1.03,1.23)	0.01	1.04 (1.01,1.07)	0.01

[a] Adjusted for year at cohort entry, ethnicity, education, smoking, baseline BMI, diabetes, family history, and aspirin/statin intake, survival model (restricted to cases) additionally adjusted for prostate cancer grade and stage.

[b] P value for trend was calculated by modeling the median of each quartile as a continuous term.

Supplemental Table 2. Odds Ratios of Association between AACs status and PSA Screening History before the 2nd Questionnaire (N=56,534)[a]

AACs status	PSA screening		OR (95% CI)
	Never	Ever	
No AACs	24655	19553	1 [reference]
AACs	6048	6278	1.27 (1.22,1.33)
No AACs	24655	19553	1 [reference]
AACs w/o med	4073	3875	1.20 (1.14,1.26)
AACs w/ med	1975	2403	1.41 (1.32,1.50)
Less than 1 year	426	468	1 [reference]
2-5 Years	551	657	1.00 (1.00,1.00)
>5 Years	806	1077	1.04 (0.87,1.24)

[a]Adjusted for age at entry, race/ethnicity, education, baseline BMI, diabetes, family history, aspirin/statin intake.

Supplemental Table 3. Hazard Ratios of Prostate Cancer Outcomes Associated with AACs Among Men with PSA Screening Information in the Multiethnic Cohort, 1998-2017 (N=56,534)[a]

		Events	Total	Minimally adjusted HR (95% CI)[b]	Fully adjusted HR (95% CI)[c]	PSA screening adjusted HR (95% CI)[d]
Incident prostate cancer	Total	5403	56534	0.95 (0.89,1.01)	0.94 (0.88,1.00)	0.93 (0.87,0.99)
	Aggressive	1697	55904	0.94 (0.84,1.05)	0.93 (0.83,1.04)	0.93 (0.83,1.03)
	Low-grade	3645	56118	0.96 (0.89,1.03)	0.94 (0.88,1.02)	0.93 (0.86,1.00)
	High-grade	1342	56118	0.90 (0.79,1.02)	0.90 (0.79,1.02)	0.89 (0.79,1.01)
	Localized	4036	55988	0.93 (0.87,1.00)	0.92 (0.86,0.99)	0.91 (0.85,0.98)
	Regional	548	55988	1.02 (0.84,1.23)	0.99 (0.82,1.20)	0.99 (0.82,1.19)
	Metastatic	273	55988	0.82 (0.62,1.10)	0.85 (0.63,1.13)	0.85 (0.64,1.14)
Prostate cancer mortality		684	56534	0.74 (0.61,0.91)	0.75 (0.61,0.92)	0.75 (0.61,0.92)
Prostate cancer survival[e]	Prostate cancer-specific mortality	543	5252	0.71 (0.57,0.90)	0.72 (0.57,0.91)	-
	All-cause death	2549	5252	0.87 (0.78,0.96)	0.88 (0.80,0.98)	-

[a]The date of 2nd questionnaire completion was used as the entry time for time-to-event analysis.

[b]Adjusted for year at cohort entry and ethnicity.

[c]Adjusted for year at cohort entry, ethnicity, education, smoking, baseline BMI, diabetes, family history, and aspirin/statin intake.

[d]Adjusted for year at cohort entry, ethnicity, education, smoking, baseline BMI, diabetes, family history, aspirin/statin intake, and PSA screening history.

[e]Additionally adjusted for prostate cancer stage and grade.
